# An Epidemiological Perspective on New Pediatric Cases of Type 1 Diabetes and Vitamin D Deficiency in South-East Romania: A Retrospective Study

**DOI:** 10.3390/children11101162

**Published:** 2024-09-25

**Authors:** Maria Ursu, Mariana Cretu-Stuparu, Gabriela Gurau, Luciana-Carmen Nitoi, Aurel Nechita, Manuela Arbune

**Affiliations:** 1 School for Doctoral Studies in Biomedical Sciences, “Dunarea de Jos” University, 800008 Galati, Romania; maria.ursu@ugal.ro; 2 “St. Ioan” Clinic Emergency Children Hospital, 800487 Galati, Romania; gabriela.gurau@ugal.ro (G.G.); aurel.nechita@ugal.ro (A.N.); 3 Medical Department, “Dunarea de Jos” University, 800008 Galati, Romania; 4 Department of Morphology and Functional Sciences, “Dunarea de Jos” University, 800008 Galati, Romania; 5 Medical Clinical Department, “Dunarea de Jos” University, 800008 Galati, Romania; luciana.nitoi@ugal.ro (L.-C.N.); manuela.arbune@ugal.ro (M.A.); 6 “St. Apostol Andrei” Clinic Emergency Hospital, 800578 Galati, Romania; 7 “St. Cuv. Parascheva” Clinic Hospital for Infectious Diseases, 800179 Galati, Romania

**Keywords:** pediatric endocrinology, metabolic disorder, type 1 diabetes, vitamin D

## Abstract

Objectives: The aim of this study is to analyze the epidemiological characteristics and the biological profile of children from the southeast of Romania who have been newly diagnosed with type 1 diabetes (T1DM) and to investigate the potential relationships between vitamin D deficiency and the onset of this disease, especially in the context of the COVID-19 pandemic. Methods: This is a retrospective study that included 79 children under the age of 18 who were diagnosed with T1DM at the St. Ioan Galati Children’s Emergency Clinical Hospital between 2018 and 2023. Their demographic data (age, sex, and home environment), medical history (family medical history, birth weight, Apgar score, and type of nutrition), and biological parameters, including glycemia, HbA1C, and vitamin D level, were collected. We used advanced statistical methods to compare the levels of vitamin D in the children with T1DM with a control group of nondiabetic children. Results: The demographic characteristics of new T1DM are a median age of 9 and female/male sex ratio of 1:3, with 50.6% living in urban areas, 59.5% with a normal body mass index, and 74.6% presenting with ketoacidosis. Vitamin D deficiency was found in 52% of diabetic cases compared to 2.53% in the nondiabetic controls. Conclusions: There is an increasing incidence of pediatric T1DM. Diabetic ketoacidosis was frequently diagnosed as an initial manifestation and has frequently accompanied lower levels of vitamin D. Children with T1DM showed significant vitamin D deficiencies compared to the control group, highlighting the need for the monitoring and supplementation of this vitamin.

## 1. Introduction

Diabetes is a complex disease involving the endocrine and metabolic systems and influenced by genetic and environmental factors. For children, these factors affect the incidence and mortality associated with diabetes differently, varying according to their geographical area [1].

Diabetes has a specific feature that delineates it from other diseases, which is that it basically implies a disorder of carbohydrate metabolism, with hyperglycemia resulting from glucose being underutilized as an energetic source and/or over-produced by inappropriate gluconeogenesis and glycogenolysis. It can be diagnosed by determining an increased concentration of glucose in venous plasma or increased hemoglobin A_1_C in the blood [2].

The prevalence of pediatric diabetes in children has increased worldwide in the last 30 years, gradually becoming a major public health problem [3]. Globally, its incidence has increased by 39.37% and, at the same time, the age of the onset of the disease has decreased [4].

Sometimes, the signs and symptoms of diabetes in children are nonspecific, contributing to a delay in the diagnostic process. Often, its first manifestations consist of hypoglycemia, hyperglycemia, or ketoacidosis, with ketoacidosis identified as the most common cause of death in diabetics [4,5].

Type 1 diabetes represents 5 to 10% of all the classifications of this disease and is a consequence of an autoimmune destruction of the pancreatic β-cells, which is revealed by the following immunological markers/antibodies: anti-islet cell, anti-glutamic acid decarboxylase (GAD), anti-insulin (IAA), anti-tyrosine phosphatases islet antigen 2 (IA2), and anti-zinc transporter 8 (ZnT8) [6].

Recently, the dysfunction of bone turnover was documented as an autoimmune feature of diabetes. Reduced bone mass has been evidenced since the early stages of type 1 diabetes; however, it could be influenced by modifiable factors, including diet and exercises, or nonmodifiable factors, such as gender, age, genetic factors, comorbidities, and drugs [7].

The interaction between glucose metabolism and bone tissue is mediated by bone-specific proteins, such as osteoprotegerin and osteocalcin. Altered insulin function is related to osteoprotegerin, which is involved in bone remodeling, while osteocalcin secretion modulates glucose tolerance [8,9].

Vitamin D is also involved in bone metabolism by regulating calcium’s absorption from the intestines and bone mineralization, and it is also recognized as an immunomodulator, exerting an influence on both the innate and adaptive immune systems. Moreover, vitamin D is related to many pathologies, such as diabetes and infectious diseases, including COVID-19 [10].

The sources of vitamin D are food and synthesis from the skin, but these are only inactive forms. After its conversion in its active form by two hydroxylation reactions, it results in calcidiol, which later binds to specific receptors and performs multiple biological roles [11]. Many scientific reports support the role of vitamin D in pancreatic beta cell function, systematic inflammation, and insulin sensitivity [12]. On the other hand, vitamin D supplements could be beneficial for children with diabetes, although the cause–effect relationship has remained indefinite until now [13].

The objectives of our study were to compare the blood levels of vitamin D in children newly diagnosed with type 1 diabetes to those in nondiabetic subjects and to describe the epidemiological, clinical, and biological features of newly diagnosed pediatric diabetic cases.

## 2. Materials and Methods

This is a retrospective study on the epidemiological characteristics and biological profile of children with type 1 diabetes that was newly diagnosed in Galati St. Ioan Children’s Clinic Emergency Hospital from January 2018 to December 2023. This hospital is the only emergency pediatric academic hospital in the southeast of Romania and, therefore, its regional morbidity indicators are relevant.

This study was designed as a piece of observational analytical research, using the hospital’s current procedures for the clinical and biological evaluation of de novo pediatric diabetes cases. The inclusion criteria of the study group were an initial presentation of type 1 diabetes patients aged under 18, with signed informed consent from their parents to allow the anonymous use of their personal data for research. We collected demographic data (age, sex, and home environment), their family’s medical history of diabetes, physiological history (birth weight and Apgar score), and history of rickets prophylaxis.

Anthropometric parameters, including weight and height, were obtained through standard measurements at the time of diagnosis, with a scale and stadiometer, being used to calculate the body mass index (BMI = Weight (kg)/Height (m)^2^). We have calculated Z-scores for BMI, according to World Health Organization child growth standards, using the “WHO Anthro Plus 1.0.4” algorithm [14]. We classified the cases as Overweight > +1SD; Obesity > +2SD; Thinness < −2SD; and Severe thinness < −3SD.

Biological profiles of the children at the time of diagnosis were evaluated by routine biological blood parameters: glycemia, HbA1c, lipid profile, proteinemia, lipase enzymes, amylase, blood electrolytes, alkaline reserve, pH, total and ionic calcium, and vitamin D levels. The laboratory evaluation of the blood samples was carried out using the VITROS 4600 or VITROS XT 7600 analyzer (QuidelOrtho Corporation, San Diego, CA, USA) by the direct spectrophotometry method for blood glucose, HbA1c, lipid profile, proteinemia, lipase, amylase enzymes, alkaline reserve, and total and ionic calcium; for the electrolytes, the potentiometry method was used. Vitamin D was measured using the YHLO-IFLASH 1800 analyzer (YHLO Biopark, Shenzhen, China) and the chemiluminescence method. pH determination was conducted with the Rapid Point 500 analyzer (Siemens Healthineers AG, Forchheim, Germany) using the Astrup method.

We categorized diabetes cases based on the presence of ketoacidosis, diagnosed using the following criteria: blood sugar higher than 200 mg/dL, blood pH lower than 7.3, blood bicarbonate lower than 18 mmol/L, and the presence of ketones in the urine. According to the consensus statement from the International Society for Pediatric and Adolescent Diabetes (ISPAD), the severity of DKA is determined by the degree of acidosis: mild (pH  <  7.3 or serum bicarbonate  <  18 mmol/L), moderate (pH  <  7.2 serum bicarbonate <  10 mmol/L), and severe (pH 7.1, serum bicarbonate  <  5 mmol/L) [15].

According to the vitamin D level, we classified the results into three categories: optimal values (30–50 ng/mL), insufficient levels (20–30 ng/mL), or deficient levels (<20 ng/mL).

The vitamin D level was compared with the values of a control group, consisting of children without diabetes or other chronic diseases, who had the same age, gender, and living environment and presented in the same period to the hospital’s emergency room. The ratio of the control group to diabetic children was 1:1.

The statistical analysis used XL-STAT version: 5 April 2022. The collected data were classified into numerical and categorical using descriptive statistical methods: mean, median, standard deviation, and frequency. The chi-square test was used to assess data distribution. We found a non-normal distribution for most variables. Comparison of the vitamin D level between the diabetic study group and the nondiabetic control group used the Mann–Whitney test. Comparison between categorial data used ANOVA, Fisher’s Exact Test, and the chi-square test for proportions A value of *p* < 0.001 was considered as significant.

This study received the institutional approval of the Children’s Emergency Clinical Hospital St. Ioan from Galati No/5982 on 7 March 2023.

## 3. Results

### 3.1. Overview of Pediatric Hospitalized Type 1 Diabetes Cases

**Figure 1:**
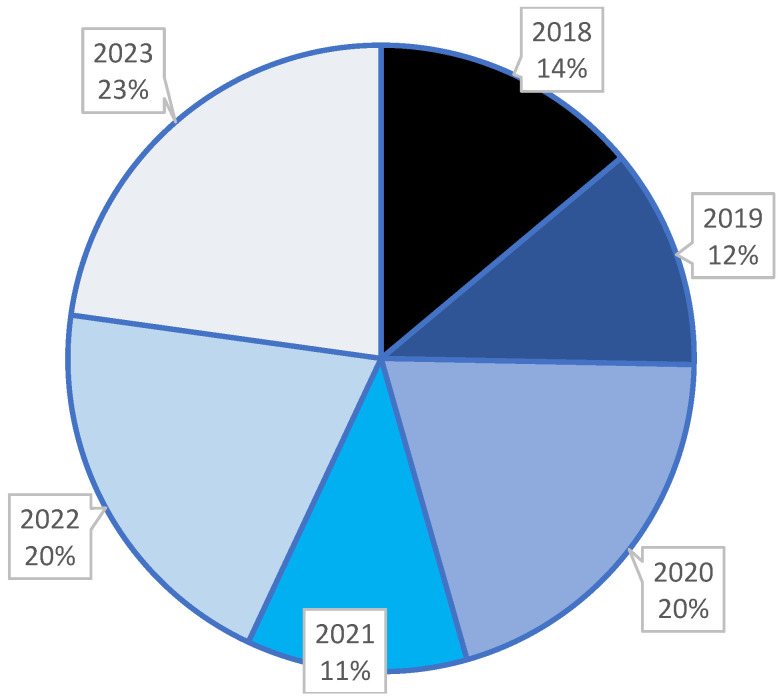
The yearly distribution of new pediatric type 1 diabetes cases in St. Ioan Children’s Clinic Hospital from Galati.

From 2018 to 2023, 79 children with newly diagnosed type 1 diabetes mellitus were identified, remarking the increased trend of the cases within the first year of the COVID-19 pandemic. The year 2021 made an exception, when the number of newly diagnosed cases decreased, probably due to the drop in the children’s addressability to the hospital, in the context of the lock-down measures (Figure 1).

**Figure 2:**
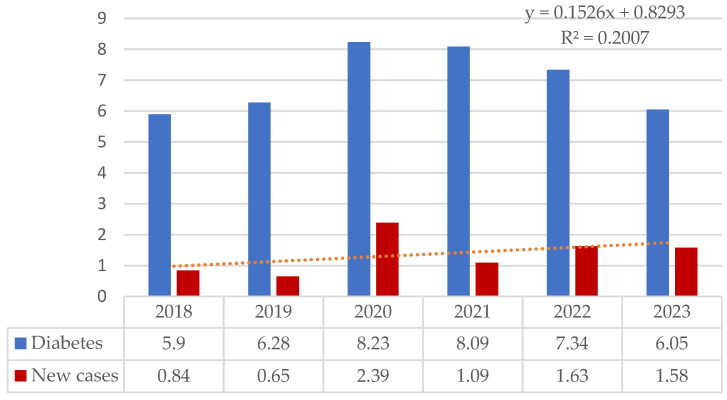
The frequency of new cases hospitalized for diabetes in Children’s Emergency Clinical Hospital from Galati from 2018 to 2023.

Among pediatric cases hospitalized with diabetes mellitus, the annual incidence varied from 0.65‰ (2019) to 2.39‰ (2020) but with a slowly increasing tendency. In the same period, the hospitalized morbidity of diabetes varied from 5.9‰ (2018) to 8.2‰ (2020) (Figure 2).

### 3.2. Demographic Characteristics of Children with New Type 1 Diabetes Diagnosis

**Figure A1:**
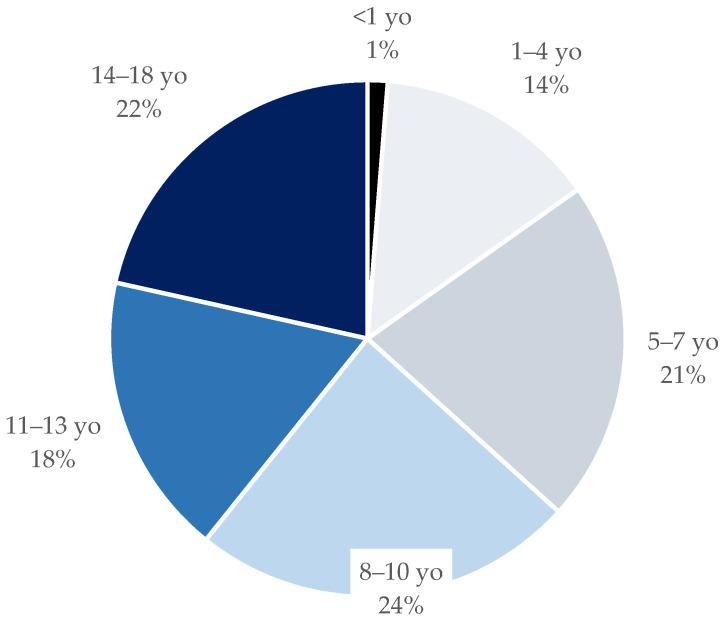
The distribution of newly diagnosed type 1 diabetes cases by age.

New cases of diabetes were identified in all age groups (Appendix A, Figure A1). The average age was 9.37 ± 4.58 years (median 9 years old), with only one case under the age of 1 year. The prepubertal age group, between 5 and 10 years old, included the most cases (45%), followed by the children between 11 and 18 years old.

The sex ratio of 1.3 highlighted the slight predominance of girls (57%).

The distribution according to the place of residence was balanced, with 50.63% living in the urban area compared to 47.94% in the rural area.

All the children of the study group were Caucasian, being representative of the regional population.

### 3.3. The Medical History of Children with New Type 1 Diabetes Diagnosis

The familial medical history was not available in 3.79% of the cases, but diabetes was noted in 32.89% of the children’s families, equally on the maternal and paternal lines, mostly, interestingly, in the grandparents (64.28%).

The physiological antecedents showed births between 36 and 41 weeks of gestational age (average of 38.92 ± 0.59 weeks), birth weight between 2000 and 4300 g (average 3180 ± 400.97 g), and Apgar score between 7 and 10 (average 8.79 ± 0.58). The diet contained breastmilk in 83.54% of the children and 93.67% had prophylaxis for rickets.

In the context of the COVID-19 pandemic, starting in 2020, children were tested for anti-SARS-CoV-2 antibodies but none had a symptomatic form of an acute infection. There were 57.63% (33/53) of cases diagnosed after the onset of the pandemic that had had IgG-positive serological markers, with negative IgM in all cases, signifying the antecedents of COVID-19 before the diagnosis of diabetes mellitus. Our study has not evidenced significant correlations between the patients with new T1DN with positive and negative serologic markers of COVID-19, related to ketoacidosis, vitamin D level, or HbA1.

### 3.4. The Nutritional Status of Children with New Type 1 Diabetes Diagnostic

**Figure 3:**
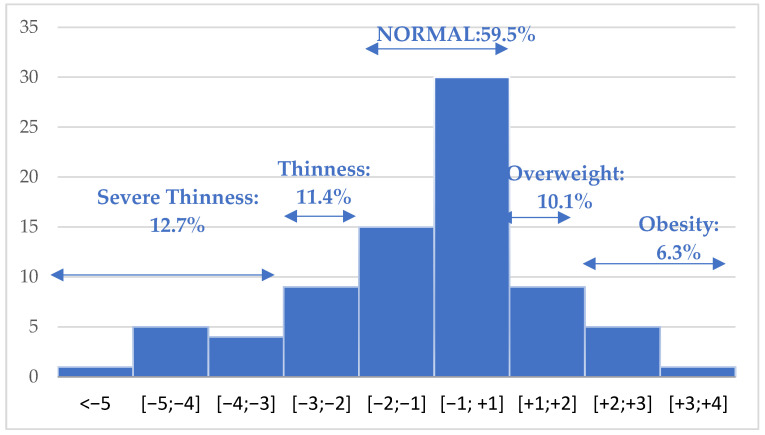
The distribution of newly diagnosed type 1 diabetes cases by Z-scores of body mass index.

The z-scores of the body mass index (BMI) adjusted by age and sex ranged from a minimum of −5.9 to a maximum of 3.9, with an average value of −0.64 ± 2.0 and a median of −0.43. The distribution of the data found 59.5% with a normal BMI, 11.4% thinness, 12.7% severe thinness, 10.1% overweight, and 6.3% obesity. We have remarked that thinness is more frequent than overweight (Figure 3).

### 3.5. The Blood Metabolic Profile of Children with New Type 1 Diabetes Diagnostic

The general metabolic profile of children with type 1 diabetes agrees with the biological changes in characteristics of ketoacidosis, the average values showing hyperglycemia 385.94 ± 168.27 mg/dL, increased HbA1 12.37 ± 2.24%, low alkaline reserve 13.09 ± 7.31, and acidosis 7.17 ± 0.34 (Table 1).

Ketoacidosis was diagnosed in 74.6% of the children with de novo diabetes, categorized by severity in 22.7% mild KA, 21.6% moderate KA, and 30.4% severe KA.

Autoimmune thyroiditis was diagnosed in 13.11% of children and hypercortisolemia in 5.08% of cases with new diabetes (Table 2).

The group of children with newly diagnosed type 1 diabetes and ketoacidosis revealed lower levels of vitamin D than the group without ketoacidosis: 20.52 ± 8.52 g/mL vs. 24.55 ± 11.71 g/mL. However, the difference was not statistically significant (Mann–Whitney test: *p* = 0.101).

### 3.6. Comparison of Vitamin D Levels in Children Newly Diagnosed with Type 1 Diabetes versus Nondiabetic Children

**Figure 4:**
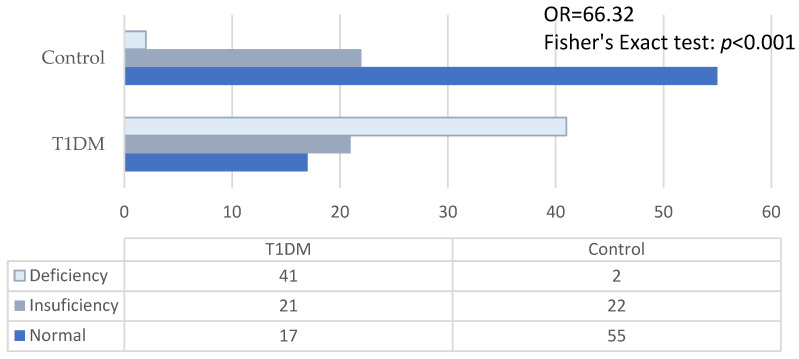
Categories of vitamin D blood levels in type 1 diabetes mellitus (T1DM) group and control group.

The level of vitamin D in children with de novo diabetes was low in 78.5% of cases, 52% having deficiencies (<20 ng/mL), compared to the group of nondiabetics, which recorded only 30.32% low values and 2.53% vitamin D deficiencies (Figure 4).

Compared to the control group, the values of vitamin D in diabetics were lower, both the average (21.45 ± 9.51 vs. 33.08 ± 9.64) and the median (19.00 vs. 31.35), the difference having statistical significance (Mann–Whitney test: *p* < 0.001).

## 4. Discussion

The role of COVID-19 as a trigger of type 1 de novo diabetes mellitus can be speculated considering the increased frequency of cases with positive SARS-CoV-2 IgG starting in 2020. Although the observation cannot be statistically confirmed in this study, numerous studies from the recent literature have been focused on this aspect. Patients with type 1 diabetes are at a higher risk of developing severe COVID-19, and the progression of diabetes is more severe when combined with SARS-CoV-2 infection. Many cases have been reported with ketoacidosis as the initial event in this context. Although the incidence of type 1 diabetes in a pandemic context has not been sufficiently investigated, the increase in incidence appears to be a plausible hypothesis, considering the immune mechanisms associated with SARS-CoV-2 infection [16,17]. Therefore, the impact of the COVID-19 pandemic on the incidence of type 1 diabetes in children is still a matter of debate [18,19].

In Romania, medical care for diabetes is carried out in a centralized system. Since 1996, the Romanian National Organization for the Protection of Children and Adolescents with Diabetes (ONROCAD) has contributed to the development of the Romanian National Diabetes Register. The register shows an increased incidence of 16.9% for type 1 diabetes in the age group 0–14 years, compared to the period from 2015 to 2019, when the increase was only 0.8% [20]. A previous study on diabetic children from the same hospital, conducted between 2010 and 2013, found that the most prevalent age group was 6–12 years old. In comparison, our study found that the most prevalent age group was 5-10 years old, suggesting a decrease in the age of type 1 diabetes diagnosis [21].

Diabetic ketoacidosis may occur in people with undiagnosed diabetes or due to poor disease management. The incidence of ketoacidosis at the onset of type 1 diabetes in high-income countries ranges from 30% to 40% [22,23].

The INNODIA Natural History Study is a multicenter study conducted between 2016 and 2021 in 18 of the most important diabetes clinics from centers in Europe. The study collected data from 673 children, adolescents, and young adults who were newly diagnosed with type 1 diabetes. The prevalence of ketoacidosis was 36%, with the most affected age group being 10–14 years old (44%) [24]. An Italian study between 2017 and 2021 found clinical criteria for ketoacidosis in 51.5% of children and adolescents newly diagnosed with type 1 diabetes, from which 18.4% were severe forms, mentioning that most of them belonged to immigrants, ethnic minorities, and impoverished populations [25].

Comparative data from our study showed a significantly higher frequency of overall ketoacidosis (75%) and severe forms (30%), possibly influenced by the COVID-19 pandemic context and potential undiagnosed COVID-19 infections [26].

Hyperglycemia could alter the activity of the hypothalamus–pituitary–adrenal axis, which is implicated in autoimmune disorders related to diabetes mellitus [27].

Krzewska et al. 2016 showed that the most common autoimmune disease associated with type I diabetes is autoimmune thyroiditis in 15%–30% of cases [28]. Similarly, in our study, abnormal values of the thyroid hormones were found in 15.18% (12 children).

Lower nocturnal cortisol metabolism in prepubertal children and other related metabolic, endocrinologic, or inflammatory diseases are explained by the brain involvement and disturbance of the hypothalamus–pituitary–adrenal axis related to juvenile type 1 diabetes mellitus [27,29].

The role of vitamin D in type 1 diabetes mellitus is controversial. A meta-analysis that included 45 studies and 6995 patients from 25 countries found 45% of cases with vitamin D deficiency, while we found over 50%. The stratified analysis highlighted that vitamin D deficiency varies depending on the year of the study, the season of the study, and the geographical region [30].

As an immunomodulator, vitamin D has become a research topic for the onset of diabetes and the evolution of complications. Recent results show that vitamin D delays the onset of diabetes and complications, intervening through different mechanisms as follows: it decreases oxidative stress, inhibits apoptosis, increases calcium influx, stimulates insulin secretion, and decreases insulin resistance. Consequently, prevention and correction of vitamin D deficiency is very necessary for diabetic patients [31].

Further on how vitamin D impacts glucose metabolism and islet cell function may be described in several pathways:

Vitamin D Receptor (VDR) Activation

Vitamin D binds to its receptor (VDR), which is expressed in pancreatic beta cells, as well as in other tissues involved in glucose metabolism, like muscle and adipose tissue. The binding of the active form of vitamin D, 1,25-dihydroxyvitamin D (1,25(OH)_2_D), to VDR leads to transcriptional regulation of genes that are critical for insulin production and secretion [12,32];

Beta-Cell Preservation and Insulin Secretion

Activation of VDR in beta cells enhances their survival and function. It does so by modulating the transcription of genes involved in insulin synthesis, secretion, and beta-cell integrity. For instance, VDR activation boosts the expression of insulin and insulin-like growth factors, which are crucial for maintaining normal glucose levels [32];

Anti-Inflammatory Effects

Vitamin D exerts anti-inflammatory effects that help reduce the risk of beta-cell destruction, which is common in autoimmune conditions like type 1 diabetes (T1D). By reducing inflammation and suppressing autoimmune attacks on beta cells, vitamin D can help preserve these cells, which are essential for insulin production [12,33];

Insulin Sensitivity

In peripheral tissues like muscles and the liver, vitamin D enhances insulin sensitivity. This occurs by increasing the expression of insulin receptors and improving the function of glucose transporters, which helps in efficient glucose uptake from the bloodstream. In this way, vitamin D modulates glucose metabolism and reduces insulin resistance, a key factor in type 2 diabetes (T2D) [12];

Modulation of Calcium Homeostasis

Vitamin D plays a role in calcium metabolism, which indirectly affects insulin secretion. Calcium is necessary for the exocytosis of insulin granules from beta cells. Vitamin D ensures proper calcium levels within the beta cells, facilitating effective insulin secretion [33,34].

A recent study from Romania assessed the levels of 25 (OH)D in the serum of children aged 2 to 18 upon admission to a university teaching hospital. The study found that 27% of the children had vitamin D deficiency, with variations based on gender and age group, occurring more frequently than in our control group. In comparison to this deficiency rate, the diabetic children in our study had almost twice the frequency of deficiency but no significant differences were found based on sex and age [35].

A retrospective study of diabetic children from Constanta, Romania, found that 63% of cases had insufficient levels of vitamin D and 20%. In our patients, the deficiency was even more common. The frequency of thyroiditis was 9.1%, which aligns with our results [36].

A study in Sudan revealed that vitamin D deficiency was prevalent among type 1 diabetes patients, and supplementation led to a significant decrease in fasting blood glucose, though changes in HbA1c and insulin needs were not significant. Further high-quality research is necessary to confirm these findings [37].

A systematic review assessed the impact of vitamin D supplementation on glycemic control in children and adolescents with type 1 diabetes mellitus and, despite the many studies reviewed, only 10 met the selection criteria, with significant variation in study design, vitamin D dosage, and duration of supplementation. The results showed mixed outcomes, with 50% of the studies indicating a significant improvement in glycemic control, the evidence remaining inconsistent [38].

Another systematic review indicates that vitamin D deficiency is also prevalent among children and adolescents with type 1 diabetes and may contribute to its development, particularly in those with genetic predispositions. While vitamin D supplementation has shown some potential in improving glycemic control and reducing fasting blood glucose levels, it remains to clarify the role of vitamin D in both the onset and management of T1DM in young populations [39].

Future perspectives of vitamin D research should evaluate the post-medication active serum level depending on variable forms of vitamin D supplements, the individual response, and the associated comorbidities in order to develop personalized health interventions [31].

### Limitations of the Study

This study’s retrospective nature prevented the systematic collection of medical history and laboratory data. As a result, we were unable to analyze the frequency of association between autoimmune diseases and the progression of diabetes. Additionally, the limited number of cases may have restricted the statistical analysis’s power.

## 5. Conclusions

The number of newly diagnosed type 1 diabetes cases is increasing among children in the South-East Romania. It is unclear whether the emergent COVID-19 pandemic has contributed to this rise. Juvenile type 1 diabetes affects children of all ages. Our study confirmed that ketoacidosis is the main initial manifestation of type 1 de novo diabetes. Children with type 1 de novo diabetes had severe vitamin D deficiencies, significantly higher compared to nondiabetic children. According to local protocols, it is necessary to administer vitamin D supplements to diabetic children in the Galati region, but additional studies are needed to evaluate the post-medication effectiveness.

The incidence of type 1 diabetes in children from the South-East Romania has been increasing, especially after the emergence of the COVID-19 pandemic.

## Figures and Tables

**Table 1:** Characteristics of the blood biochemistry specific in newly diagnosed children with diabetes type 1.

	Normal Values	Average ± SD	Median	Max	Min
Glycaemia	75–150 mg/dL	377.63 ± 165.23	362	1367	131
HbA1	4–6%	12.39 ± 2.19	12.16	20.58	6.81
pH	7.35–7.45	7.18 ± 0.33	7.25	9	6
Na_2_CO_3_	22–30 mmol/L	13.11 ± 7.44	13	30	5
Na^+^	135–45 mmol/L	136.11 ± 4.45	136.4	158.5	123
K^+^	3.6–4.8 mmol/L	4.15 ± 0.52	4.16	5.41	2.54
Cl^−^	95–105 mmol/L	102.55 ± 5.93	102	125	89
Total Calcium	8.9–10.7 mg/dL	9.78 ± 0.66	9.8	11.4	7.7
Ionic Calcium	4.2–5.2 mg/dL	4.18 ± 0.40	4.20	5.73	3

**Table 2:** Endocrinological markers associated with newly diagnosed DM in children.

	N *	NV	Average ± SD	Median	% NV	% Over NV	% Under NV
Cortisol	59	5–25 μ/dL	15.34 ± 10.0	15.1	91.5%	5.0%	3.4%
ATPO	61	0–35 IU/mL	69.93 ± 253.4	10	86.9%	13.1%	-
T3	57	2.7–5.2 pg/mL	2.89 ± 1.17	2.78	94.7%	5.2%	-
TSH	60	0.6–4.84 mU/L	2.25 ± 1.5	1.83	92.4%	4.5%	3.0%
Vitamin D	73	30–50 ng/mL	21.54 ± 9.51	19	20.5%	-	79.4%

* N: number of cases; NV: normal values; SD: standard deviation.

## Data Availability

The raw data supporting the conclusions of this article will be made available by the authors on request.
